# Cross-Platform Detection of Psychiatric Hospitalization via Social Media Data: Comparison Study

**DOI:** 10.2196/39747

**Published:** 2022-12-30

**Authors:** Viet Cuong Nguyen, Nathaniel Lu, John M Kane, Michael L Birnbaum, Munmun De Choudhury

**Affiliations:** 1 School of Interactive Computing Georgia Institute of Technology Atlanta, GA United States; 2 Department of Psychiatry The Zucker Hillside Hospital Northwell Health Glen Oaks, NY United States; 3 The Feinstein Institute for Medical Research Northwell Health Manhasset, NY United States; 4 The Donald and Barbara Zucker School of Medicine at Hofstra/Northwell Hempstead, NY United States

**Keywords:** schizophrenia, mental health, machine learning, clinical informatics, social media, mobile phone

## Abstract

**Background:**

Previous research has shown the feasibility of using machine learning models trained on social media data from a single platform (eg, Facebook or Twitter) to distinguish individuals either with a diagnosis of mental illness or experiencing an adverse outcome from healthy controls. However, the performance of such models on data from novel social media platforms unseen in the training data (eg, Instagram and TikTok) has not been investigated in previous literature.

**Objective:**

Our study examined the feasibility of building machine learning classifiers that can effectively predict an upcoming psychiatric hospitalization given social media data from platforms unseen in the classifiers’ training data despite the preliminary evidence on identity fragmentation on the investigated social media platforms.

**Methods:**

Windowed timeline data of patients with a diagnosis of schizophrenia spectrum disorder before a known hospitalization event and healthy controls were gathered from 3 platforms: Facebook (254/268, 94.8% of participants), Twitter (51/268, 19% of participants), and Instagram (134/268, 50% of participants). We then used a 3 × 3 combinatorial binary classification design to train machine learning classifiers and evaluate their performance on testing data from all available platforms. We further compared results from models in intraplatform experiments (ie, training and testing data belonging to the same platform) to those from models in interplatform experiments (ie, training and testing data belonging to different platforms). Finally, we used Shapley Additive Explanation values to extract the top predictive features to explain and compare the underlying constructs that predict hospitalization on each platform.

**Results:**

We found that models in intraplatform experiments on average achieved an *F*_1_-score of 0.72 (SD 0.07) in predicting a psychiatric hospitalization because of schizophrenia spectrum disorder, which is 68% higher than the average of models in interplatform experiments at an *F*_1_-score of 0.428 (SD 0.11). When investigating the key drivers for divergence in construct validities between models, an analysis of top features for the intraplatform models showed both low predictive feature overlap between the platforms and low pairwise rank correlation (<0.1) between the platforms’ top feature rankings. Furthermore, low average cosine similarity of data between platforms within participants in comparison with the same measurement on data within platforms between participants points to evidence of identity fragmentation of participants between platforms.

**Conclusions:**

We demonstrated that models built on one platform’s data to predict critical mental health treatment outcomes such as hospitalization do not generalize to another platform. In our case, this is because different social media platforms consistently reflect different segments of participants’ identities. With the changing ecosystem of social media use among different demographic groups and as web-based identities continue to become fragmented across platforms, further research on holistic approaches to harnessing these diverse data sources is required.

## Introduction

### Background

Despite its relatively low prevalence compared with other mental health disorders, the burden of schizophrenia spectrum disorder (SSD) on patients, families, and society is substantial [1]. To mitigate the burden of SSD, early diagnosis and treatment are crucial. However, psychotic disorders, including SSD, often receive delayed attention and care, resulting in worse health outcomes [2,3]. At the same time, the use of social media is high among patients with serious psychotic disorders such as SSD, especially among adolescents and young adults, when SSD typically emerges [4,5]. For instance, Birnbaum et al [4] studied social media use among adolescents and young adults with psychotic and mood disorders and found that 97.5% of participants (mean age 18.3 years) regularly used social media, spending approximately 2.6 (SD 2.5) hours per day on the web. Similarly, Miller et al [5] studied the use of digital technologies among patients diagnosed with SSD and found that, among participants with access to the internet, 98% reported using at least one social media service and 57% used social media daily.

Given this information, there has been an established body of research on using social media data to identify and predict psychiatric outcomes of social media users with SSD using machine learning classifiers [6-8]. The most robust data sources available to train these classifiers consist of textual content posted on the web. Prior work in speech and text analysis among patients with SSD has identified reliable linguistic markers associated with SSD, which have been successfully used as features for the aforementioned classifiers [7,9,10]. These include certain word frequencies, word categories, and self-referential pronouns [11,12]. Given that the use of image- and video-based social media platforms such as Instagram, Snapchat, and TikTok is associated with youths, there has also been prior work in the analysis of images comparing between patients with SSD and healthy controls [13,14]. Hänsel et al [14] identified additional image markers associated with SSD, such as the image’s colorfulness and saturation and the average number of faces per image. By exploiting these markers, previous research conducted by Birnbaum et al [15] and Ernala et al [8] built classifiers to distinguish between users with a confirmed diagnosis of SSD and healthy controls on Facebook and Twitter with area under the receiver operating characteristic curve (AUROC) scores of 0.75 and 0.82, respectively.

Although such results demonstrate the potential of automated techniques in predicting the mental health outcomes of individuals with SSD via social media data, many research gaps remain that need to be addressed before psychiatrists can reliably deploy such techniques for clinical purposes. Most prior work in this area primarily focused on a single source of social media data, either exclusively from Twitter or Facebook, for downstream classification and analysis tasks [16]. However, previous research has also shown that many social media users, especially youths, use different social media platforms for different purposes because of their variety in affordances and culture. Among youths, Facebook use is associated with keeping up with close and distant friends, whereas Instagram and Snapchat use is associated with self-expression and gratification [17,18]. In addition, researchers have argued that social media users have fragmented identities across platforms [19,20]. Therefore, using a single source of social media data to build psychiatric hospitalization prediction models may potentially lead to low-sensitivity prediction models, making them unsuitable for clinical purposes. However, few studies have quantified the extent to which classifiers trained on data from one social media platform are generalizable to other platforms. To this end, our study aimed to measure the generalizability of social media–based classifiers aimed at predicting upcoming psychiatric hospitalizations to data from unseen social media platforms. In addition, we aimed to surface any evidence of the differing fragmented identities that are reflected on 3 popular social media platforms—Twitter, Facebook, and Instagram—that might affect the models’ generalizability.

### Objectives

The research question we attempted to answer was as follows: given the preliminary evidence of fragmented identities that are reflected on the investigated social media platforms, can we build classifiers that can effectively detect users at risk of an upcoming psychiatric hospitalization using social media data from platforms unseen in the training data?

To answer our research question, we collated textual and image content (if available) from consenting participants’ social media data from Facebook, Twitter, and Instagram. We then trained platform-specific classifiers to distinguish between social media data from healthy controls and data from patients with SSD with an upcoming psychiatric hospitalization. We compared the performance of classifiers on testing data between seen and unseen social media platforms from the training data. We also compared and analyzed the top predictive features and the feature importance distributions between the 3 platform-specific classifiers, with a view toward finding potential empirical evidence for fragmented identities between the various social media platforms.

## Methods

### Recruitment

We recruited participants clinically diagnosed with SSD and clinically verified healthy controls aged between 15 and 35 years. These data were collected as part of a broader research initiative involving the authors of this paper to identify technology-based health information to provide early identification, intervention, and treatment for young adults with SSD [6].

For participants with SSD aged between 15 and 35 years (141/268, 52.6%), diagnoses were based on clinical assessment of the most recent episode and were extracted from participants’ medical records at the time of their consent. Participants in this group were recruited from the Northwell Health Zucker Hillside Hospital and collaborating institutions located in East Lansing, Michigan. Participants were excluded if they had an IQ of <70 (per clinical assessment), autism spectrum disorder, or substance-induced psychotic disorder.

In addition, healthy volunteers aged between 15 and 35 years (127/268, 47.4%) were approached and recruited from an existing database of eligible individuals who had already been screened for previous research projects at Zucker Hillside Hospital and had agreed to be recontacted for additional research opportunities. Healthy status was determined by either the Structured Clinical Interview for the Diagnostic and Statistical Manual of Mental Disorders conducted within the past 2 years or the Psychiatric Diagnostic Screening Questionnaire [21,22]. Participants were excluded if clinically significant psychiatric symptoms were identified during the screening process. Additional healthy volunteers were recruited from a southeastern university via a web-based student community research recruitment site. Finally, healthy volunteers were also recruited from the collaborating institutions located in East Lansing, Michigan.

### Data Collection

All consenting participants were asked to download and share their Facebook, Twitter, and Instagram data archives. We collected all linguistic content from participants’ Facebook and Twitter archives (ie, status updates and comments on Facebook and posts shared on Twitter). In addition, we collected image content from participants’ Facebook and Instagram archives, including profile pictures and story photos.

Next, we also collected the medical history of each participant (following consent and adoption of Health Insurance Portability and Accountability Act–compliant policies). This included primary and secondary diagnosis codes, the total number of hospitalizations, and admission and discharge dates for each hospitalization event. Hospitalization data were collected from the medical records at the time of consent. As all consented patient participants in the study had also received care at the Zucker Hillside Hospital, the medical records at the hospital were accurate and up to date to the best of the hospital’s efforts. We only counted psychiatric hospitalizations (not hospitalizations for other nonpsychiatric reasons). Thereafter, the study team accessed the corresponding consented patients’ medical records to extract all their recorded hospitalization events in a similar manner to previous studies using this source of data [6,23].

**Figure 1:**
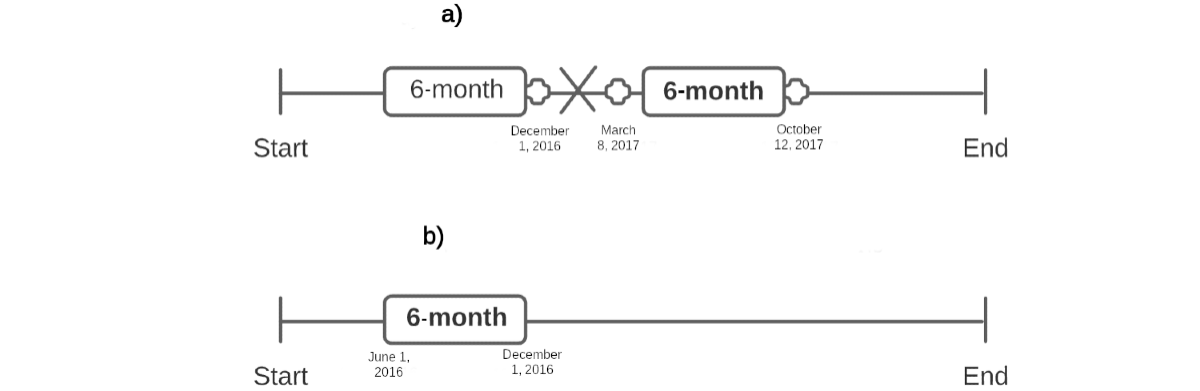
Diagram representing the windowing process used to gather participants’ social media data before hospitalization events. Bold text represents the selected data windows. Crosses represent hospitalization events. The X represents invalid data windows. A: Windowing—with hospitalizations; B: Windowing—without hospitalizations.

Finally, we collected social media data from all available platforms for each participant with at least one known hospitalization event within a 6-month window before the latest hospitalization event, ensuring that there were no hospitalization events within these 6 months. This was done to ensure that the data gathered were representative of the participants’ healthy mental status before symptomatic exacerbation and subsequent hospitalization. A 6-month period, which we refer to as the *windowed data*, was selected as it represents an interval of time long enough to identify changes signaling symptomatic exacerbation while also containing sufficient data required to train machine learning models. For healthy control participants without any hospitalizations, we randomly sampled a nonempty 6-month window of social media data for each available social media platform (nonempty meaning that there was at least some social media activity). Figure 1 provides a visual description of the windowing process.

### Feature Engineering

To encode participants’ social media data for the downstream classification and analysis tasks outlined in our research objectives, we identified and extracted the following categories of features from these data for all 3 investigated social media platforms: (1) n-gram language features (n=500), (2) Linguistic Inquiry and Word Count (n=78), (3) lexico-semantic features (n=3), (4) activity features (n=9), and (5) image features (n=23; Instagram and Facebook only).

The specific feature categories were chosen based on relevant previous literature, particularly relating to the use of social media data to infer mental health attributes and psychiatric outcomes [7,8]. Note that all features were computed at the individual participant level. More details about this process can be found in Multimedia Appendix 1 [7,12,14,24-29].

### Feature Selection

Using the aforementioned features, for each of the 3 examined social media platforms, we encoded available participants’ textual and image data on Facebook and Instagram into 613-dimensional feature vectors and textual data on Twitter into 590-dimensional feature vectors. This yielded a Facebook data set of dimension 254 × 613, a Twitter data set of dimension 51 × 590, and an Instagram data set of dimension 134 × 613. We shall refer to these data sets as F, T, and I for Facebook, Twitter, and Instagram, respectively.

As the feature set might contain features that are noisy and irrelevant, the classification models may be unstable and produce suboptimal results [30]. To maximize the predictive power of the models while also reducing the redundancy and computational resources needed to train them, feature selection methods were used [30]. More specifically, we adopted the ANOVA *F* test to rank the features based on their *F* statistic under the test, which has been shown to produce optimal feature sets in previous research on the classification of social media data belonging to patients with SSD [8,11].

We trained a random forest model, with 5-fold stratified cross-validation to fine-tune hyperparameters, on data sets F, T, and I with an 80:20 train-test split, using only the top *k* percent of features based on the ranking given by the ANOVA *F* test on the classification, where *k* is between 10 and 100 in increments of 10. Via an examination of the evaluation metrics on the test sets (described in the Classification Algorithms and Metrics section), we determined that using only the top 20% of the features (based on their *F* statistic under the ANOVA *F* test) yielded the best results on unseen data across all 3 platforms. We will be using this subset of features moving forward.

### Combinatorial Classification Methods

**Figure 2:**
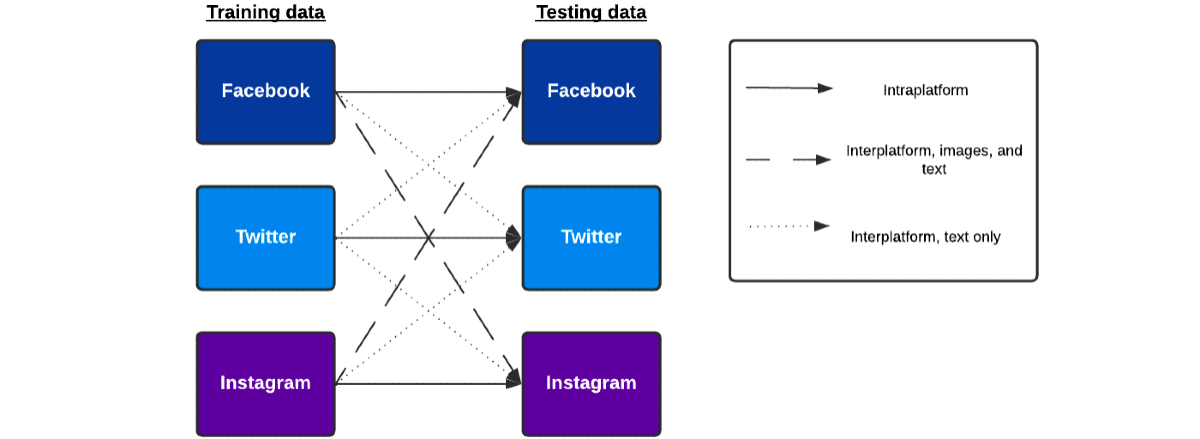
Diagram representing the classification experiments performed and their nature within the 3 × 3 combinatorial design.

To answer the research question laid out in the Introduction section, we adopted a 3 × 3 combinatorial classification design, where we trained and tested machine learning models on the psychiatric hospitalization prediction task using all possible pairs of training and testing data sets. Figure 2 provides a visual description of our experimental design. For intraplatform experiments (where the training and testing data came from the same platform; eg, training and testing on Facebook data), we trained and tested the models on an 80 to 20 train-test label-stratified split based on the Scikit-learn *train_test_split()* function (version 0.24.1) [31]. For interplatform experiments (where the training and testing data came from different platforms; eg, training on Facebook data and testing on Instagram data), we trained the model on the entirety of the training data set and evaluated it on the entirety of the testing data set.

### Classification Algorithms and Metrics

For both intra- and interplatform experiments, training data represented by the top 20% of features (as described in the Feature Selection section) were fed into a model to learn the classification task. We tried training the model over several algorithms, including random forest, logistic regression, support vector machine, and multilayer perceptron [32]. We selected these algorithms as they represented a variety of different types of learning algorithms [32]. This ensured that our analysis of performance differences between intra- and interplatform experiments would hold irrespective of the learning algorithm selection. We used the Scikit-learn implementation (version 0.24.1) for all the aforementioned algorithms [31]. For each algorithm, we fine-tuned its hyperparameters using 5-fold stratified cross-validation via the Scikit-learn *GridSearchCV()* pipeline, retaining the best hyperparameters per algorithm for analysis [31]. The chosen hyperparameters for each classification algorithm are provided in Textbox 1 (all other hyperparameters were left as default according to the Scikit-learn specification).

We measured the performance of the models using the metrics outlined in Textbox 2, all of which are commonly used in binary classification models. In this case, we abbreviated the number of true positives, true negatives, false positives, and false negatives as TP, TN, FP, and FN, respectively [33].

**Hyperparameters chosen for each classification algorithm.**

**Random forest**

- max_depth: 15
- n_estimators: 100
- max_features: none

**Logistic regression**

- Penalty: l2
- C: 0.1

**Support vector machine**

- Kernel: rbf
- C: 0.01
- Gamma: scale

**Multilayer perceptron**

- Alpha: 0.0001
- Hidden_layer_sizes: (512, 256, 128)

**Metrics used to measure model performance.**

**Accuracy**

- Also known as Rand accuracy, the ratio of correct predictions to all predictions

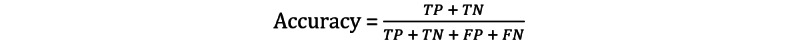

**Precision**

- The ratio of correct positive predictions to the total number of positive predictions

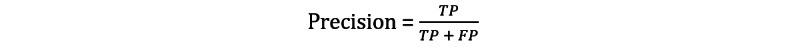

**Recall**

- The ratio of correct positive predictions to the total number of true positive instances

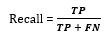

***F*_1_-score**

- The harmonic mean between precision and recall

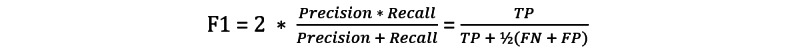

**Area under the receiver operating characteristic curve (AUROC)**

- The AUROC, which plots the false positive rate against the true positive rate and, in practice, is often estimated using the trapezoidal rule with the following formula:

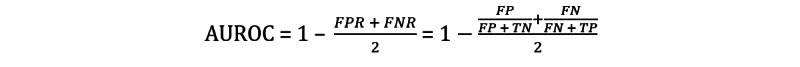

### Feature Importance Selection

We used Shapley Additive Explanations (SHAP) to examine how certain features affected our model’s decision to predict users with potential psychiatric hospitalization because of SSD given their social media data from the 3 inspected social media platforms. Our decision to use SHAP rather than other explainability methods stems from the fact that SHAP is not only model-agnostic but also the most theoretically sound explainability framework among the available options. This is because SHAP feature scores can be calculated for localized samples and for the entire global data set [34]. SHAP is based on Shapley values, a game-theoretical concept that intuitively describes each feature’s contribution to the outcome after considering all possible combinations of features [35].

For each of the intraplatform experiments within the 3 × 3 combinatorial design and each machine learning model, we calculated the average SHAP values for each of the features (ie, their importance to the prediction) across all instances within the testing set. We then recorded the list of features sorted in descending order according to the average SHAP values measured by each model. In the case of models with native support for feature importance extraction, including random forest (Gini importance) and logistic regression (feature coefficients), we also calculated and recorded them in an equivalent manner to SHAP values.

### Robustness Checks

To ensure that our findings regarding differences in model performance between models and between intra- and interplatform experiments still held when certain aspects of the training and testing data sets were made more ideal, we performed several robustness checks, which are described in Multimedia Appendix 1.

### Ethics Approval

The study was approved by the institutional review board of Northwell Health (the coordinating institution) and the institutional review board of the participating partners (Georgia Tech approval H21403). Participants were recruited from June 23, 2016, to December 4, 2020. Written informed consent was obtained from adult participants and legal guardians of participants aged <18 years. Assent was obtained from participating minors.

## Results

### Data Characteristics

**Figure 3:**
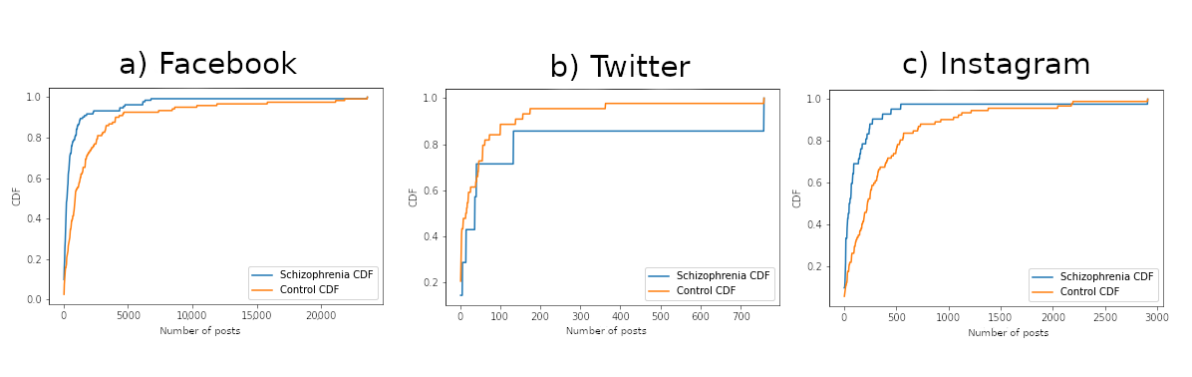
Cumulative distribution function (CDF) curves of users and their number of posts for the schizophrenia spectrum disorder and control classes per data set: (A) Facebook (left), (B) Twitter (center), and (C) Instagram (right).

In total, 268 participants (mean age 24.73, SD 5.64 years; male: 127/268, 47.4%; SSD: 141/268, 52.6%) with nonempty windowed data for at least one platform were included. Of these 268 participants, 254 (94.8%; SSD: 133/254, 52.4%) had valid windowed Facebook data, 51 (19%; SSD: 7/51, 13.7%) had valid windowed Twitter data, and 134 (50%; SSD: 42/134, 31.3%) had valid windowed Instagram data. Among participants with valid data for more than one platform, 17.5% (47/268; SSD: 5/47, 10.6%) had valid data for both Facebook and Twitter, 14.2% (38/268; SSD: 4/38, 10.5%) had valid data for both Twitter and Instagram, and 44.4% (119/268; SSD: 34/119, 28.6%) had valid data for both Facebook and Instagram. Finally, 14.2% (38/268; SSD: 4/38, 10.5%) of participants had valid data for all 3 platforms. Table 1 shows the demographic and clinical characteristics of these 268 participants. Table 2 describes the summary statistics, including mean and median, for these windowed data for each of the 3 social media platforms grouped by clinical status (SSD vs control). Figure 3 shows the distribution of available posts for participants in each of the 3 investigated platforms.

**Table 1:** Demographic and clinical characteristics of the participants (N=268).

Characteristic			SSD^a^ (n=141)		Control (n=127)		Full sample
Age (years), mean (SD)			24.86 (5.49)		24.57 (5.82)		24.73 (5.64)
**Sex, n (%)**							
	Male	89 (63.1)		38 (29.9)		127 (47.4)	
	Female	52 (36.9)		89 (70.1)		141 (52.6)	
**Race or ethnicity, n (%)**							
	African American or Black	64 (45.4)		19 (15)		83 (31)	
	Asian	20 (14.2)		23 (18.1)		43 (16)	
	White	37 (26.2)		75 (59.1)		112 (41.8)	
	Mixed race or other	15 (10.6)		5 (3.9)		20 (7.5)	
	Hispanic	5 (3.5)		4 (3.1)		9 (3.4)	
	Pacific Islander	0 (0)		1 (0.8)		1 (0.4)	
**Primary diagnosis, n (%)**							
	Schizophrenia	67 (47.5)		N/A^b^		67 (25)	
	Schizophreniform	26 (18.4)		N/A		26 (9.7)	
	Schizoaffective	25 (17.7)		N/A		25 (9.3)	
	Unspecified SSDs	23 (16.3)		N/A		23 (8.6)	
	No diagnosis	N/A		127 (100)		127 (47.4)	

^a^SSD: schizophrenia spectrum disorder.

^b^N/A: not applicable.

**Table 2:** Summary statistics for windowed data for both the control class and the schizophrenia spectrum disorder (SSD) class (ie, participants hospitalized with SSD). In this table, we consider data from Facebook, Twitter, and Instagram, as mentioned previously.

	Facebook (user: n=254; post: n=169,425)			Twitter (user: n=51; post: n=23,777)			Instagram (user: n=134; post: n=23,551)	
	SSD class	Control class	SSD class		Control class	SSD class		Control class
Total users, n (%)	133 (52)	121 (48)	7 (14)		44 (86)	42 (31)		92 (69)
Total posts, n (%)	114,793 (68)	54,632 (32)	991 (4)		22,786 (96)	7111 (30)		16,440 (70)
Posts, mean (SD)	863.1 (2365.1)	451.5 (818.87)	141.6 (255)		519.9 (1166.9)	169.3 (445.4)		178.7 (234.6)
Posts, median	260	184	37		138	54.5		103
Posts, range	2-23,589	1-4852	1-758		1-7056	1-2909		1-1328

### Results of Combinatorial Classification

**Figure 4:**
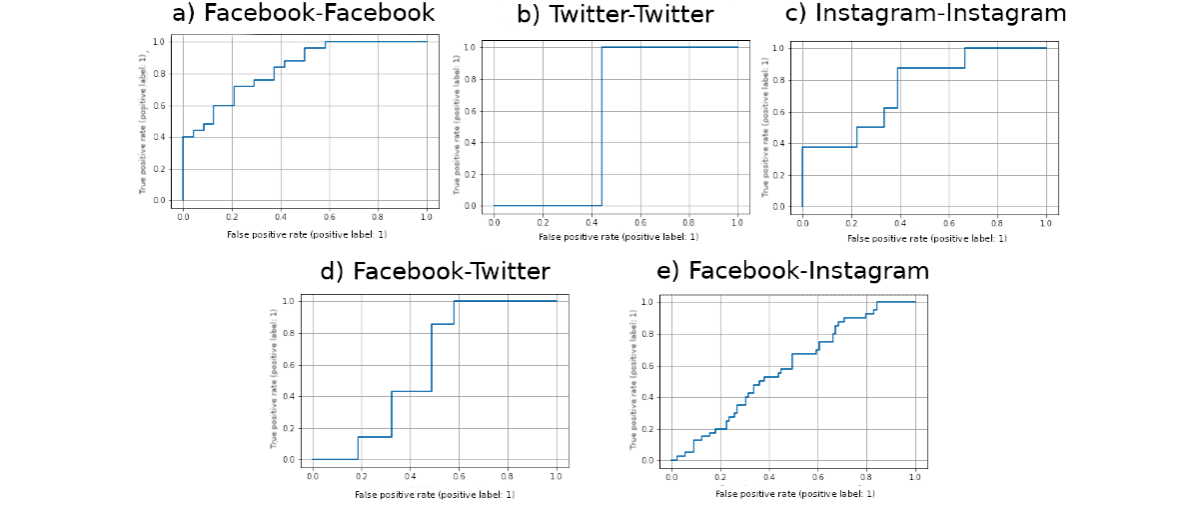
Receiver operating characteristic (ROC) curves for the classification experiments given the best logistic regression model. (A), (B), and (C) are curves for the Facebook, Twitter, and Instagram intraplatform results, respectively, from Table 3. (D) and (E) are the ROC curves for the interplatform experiments from Table 4, where Facebook was used as the training data.

We report the full results of the intraplatform experiments in Table 3. We also report the full results of the interplatform experiments in Tables 4 to 6. Finally, we report the receiver operating characteristic curves for the best-performing logistic regression model for the experiments from Tables 3 to 6 in Figure 4.

Elaborating on the results from Table 3, we found that, among the 4 classification algorithms that we used, the logistic regression model performed the best across the 3 intraplatform experiments, with the best performances for all of them. More elaborately, for the intraplatform experiments, performance reached its peak with the logistic regression model with an average *F*_1_-score of 0.72 (SD 0.07), accuracy of 0.81 (SD 0.08), and AUROC of 0.749 (SD 0.06). In contrast, the worst-performing model (in this case, multilayer perceptron) achieved an average *F*_1_-score of 0.521 (SD 0.19), accuracy of 0.714 (SD 0.19), and AUROC of 0.623 (SD 0.16) for the intraplatform experiments. Thus, we will be using the logistic regression model for further analysis regarding feature importance between platforms. These results align with previous research and, thus, could be considered a soft replication of those findings [8,15].

By contrast, by aggregating the metrics for the interplatform experiments presented in Tables 4 to 6, the average *F*_1_-score decreased to 0.428 (SD 0.11), accuracy decreased to 0.559 (SD 0.06), and AUROC decreased to 0.533 (SD 0.03) for the logistic regression model. This constitutes, on average, a drop of 40%, 31.4%, and 28.8% in *F*_1_-score, accuracy, and AUROC score, respectively, from the intraplatform experiments. As just demonstrated, when comparing the effectiveness of models between intraplatform and interplatform experiments, we found a consistent drop in performance for all the investigated social media platforms. The drop in test *F*_1_-score, given the best-performing logistic regression model, was the most drastic for Facebook at 0.364 (46%) and least drastic for Twitter at 0.08 (14%), averaging a drop of 0.285 (40%, SD 0.13) going from 0.713 for intraplatform experiments to 0.428 for interplatform experiments. Such trends hold even when disparities in data set size and dual-platform data availability (as described in the Methods section under Robustness Checks) are applied to the training and testing data (Multimedia Appendix 1).

**Table 3:** Classification results for all intraplatform classification experiments. In this table, for instance, Facebook indicates the Facebook-Facebook experiment.

Model	Facebook						Twitter						Instagram				
	Acc^a^	P^b^	R^c^	*F*_1_	AUROC^d^	Acc		P	R	*F*_1_	AUROC	Acc		P	R	*F*_1_	AUROC
Random forest	0.739	0.739	0.738	0.738	0.709	0.745		0.150	0.116	0.116	0.494	0.7		0.648	0.637	0.637	0.681
SVM^e^	0.722	0.747	0.692	0.715	0.723	0.854		0.541	0.45	0.463	0.697	0.740		0.737	0.757	0.743	0.805
MLP^f^	0.506	0.406	0.507	0.367	0.516	0.845		0.458	0.45	0.426	0.692	0.792		0.771	0.794	0.77	0.840
Logistic regression	0.759	0.767	0.758	0.756	0.727	0.881		0.742	0.6	0.63	0.772	0.792		0.771	0.801	0.773	0.848

^a^Acc: accuracy.

^b^P: precision.

^c^R: recall.

^d^AUROC: area under the receiver operating characteristic curve.

^e^SVM: support vector machine.

^f^MLP: multilayer perceptron.

**Table 4:** Classification results for the interplatform classification experiments for Facebook training data.

Model	Twitter						Instagram				
	Acc^a^	P^b^	R^c^	*F*_1_	AUROC^d^	Acc		P	R	*F*_1_	AUROC
Random forest	0.392	0.221	0.88	0.354	0.579	0.379		0.328	0.952	0.488	0.537
SVM^e^	0.545	0.253	0.72	0.373	0.612	0.432		0.337	0.860	0.483	0.550
MLP^f^	0.587	0.240	0.55	0.334	0.573	0.435		0.332	0.812	0.471	0.539
Logistic regression	0.628	0.246	0.47	0.323	0.567	0.472		0.344	0.775	0.476	0.555

^a^Acc: accuracy.

^b^P: precision.

^c^R: recall.

^d^AUROC: area under the receiver operating characteristic curve.

^e^SVM: support vector machine.

^f^MLP: multilayer perceptron.

**Table 5:** Classification results for the interplatform classification experiments for Twitter training data.

Model	Facebook						Instagram				
	Acc^a^	P^b^	R^c^	*F*_1_	AUROC^d^	Acc		P	R	*F*_1_	AUROC
Random forest	0.531	0.569	0.378	0.452	0.536	0.628		0.331	0.207	0.252	0.512
SVM^e^	0.514	0.53	0.537	0.530	0.513	0.563		0.340	0.42	0.373	0.523
MLP^f^	0.533	0.561	0.440	0.492	0.536	0.557		0.325	0.395	0.356	0.512
Logistic regression	0.534	0.552	0.522	0.535	0.535	0.578		0.362	0.47	0.408	0.548

^a^Acc: accuracy.

^b^P: precision.

^c^R: recall.

^d^AUROC: area under the receiver operating characteristic curve.

^e^SVM: support vector machine.

^f^MLP: multilayer perceptron.

**Table 6:** Classification results for the interplatform classification experiments for Instagram training data.

Model	Facebook						Twitter				
	Acc^a^	P^b^	R^c^	*F*_1_	AUROC^d^	Acc		P	R	*F*_1_	AUROC
Random forest	0.51	0.523	0.612	0.563	0.507	0.751		0.369	0.42	0.386	0.624
SVM^e^	0.524	0.544	0.51	0.524	0.525	0.691		0.213	0.25	0.229	0.521
MLP^f^	0.554	0.584	0.48	0.526	0.557	0.683		0.201	0.23	0.214	0.51
Logistic regression	0.516	0.524	0.689	0.595	0.51	0.628		0.256	0.52	0.342	0.587

^a^Acc: accuracy.

^b^P: precision.

^c^R: recall.

^d^AUROC: area under the receiver operating characteristic curve.

^e^SVM: support vector machine.

^f^MLP: multilayer perceptron.

### Feature Importance Analysis

We hypothesized that the decrease in performance from intraplatform experiments to interplatform experiments, as presented previously, was driven by differences in feature importance learned by models when trained on data from different social media platforms (even when they shared the same feature set). By extracting the list of SHAP features from the models per the method described previously, we found support for this hypothesis. Specifically, we observed little overlap between them across platforms among the top 25 features for each model and platform (when holding the model constant). On average, there were only 4.66 overlapping features for the same logistic regression classification model across platforms (the best-performing model based on the previous discussions). In addition, we found that the lists of feature importance for each of the platforms, based on the logistic regression model, had very weak rank correlation pairwise. Fully elaborating on the statistical results for the Kendall rank correlation coefficient, we found very weak rank correlations between the ranked lists of feature importance for Facebook and Twitter (τ_b_=0.081; *P*=.003), Facebook and Instagram (τ_b_=0.041; *P*=.01), and Twitter and Instagram (τ_b_=0.055; *P*=.05). We report the average SHAP values and logistic regression coefficients of the top 10 features based on their SHAP values, along with their average value in the SSD class and the control class, in Table 7.

**Table 7:** Top 10 features for the logistic regression (LR) model for each of the platforms (Linguistic Inquiry and Word Count features are italicized) based on their Shapley Additive Explanations (SHAP) values.

Platform and feature acronym		Feature description		SHAP value		LR coefficient		SSD^a^ group average (SD)		Control group average (SD)
**Facebook**										
	Avg_post_readability	Average post readability, as measured using the SMOG^b^ index	0.761		−0.268		5.6341 (2.74)		6.8048 (1.92)	
	*Quant*	Ratio of words within the “quantifiers” category	0.4195		−0.189		0.0012 (0.0012)		0.0016 (0.0012)	
	*Negemo*	Ratio of words within the “negative emotions” category	0.0953		0.244		0.0043 (0.0035)		0.0031 (0.0022)	
	*Money*	Ratio of words within the “money” category	0.0739		−0.216		0.0007 (0.001)		0.0011 (0.002)	
	*Swear*	Ratio of words within the “swear” category	0.0628		0.236		0.0017 (0.0025)		0.0007 (0.001)	
	Ratio_octile8	Ratio of activities from 9 PM to midnight	0.0443		0.077		0.1443 (0.149)		0.1241 (0.158)	
	Ratio_octile7	Ratio of activities from 6 PM to 9 PM	0.0409		0.177		0.1561 (0.1745)		0.1054 (0.125)	
	*Anger*	Ratio of words within the “anger” category	0.0095		0.191		0.0018 (0.002)		0.0009 (0.001)	
	Dream	Ratio of “dream” within the overall bag of words	0.0077		0.224		0.2028 (0.468)		0.0746 (0.24)	
	Fun	Ratio of “fun” within the overall bag of words	0.0043		−0.209		0.5722 (1.19)		1.1315 (1.76)	
**Twitter**										
	*Conj*	Ratio of words within the “conjunctions” category	0.2319		−0.063		0.0001 (0.0002)		0.0003 (0.0004)	
	*Adj*	Ratio of words within the “adjectives” category	0.1825		−0.05		0.0057 (0.004)		0.0080 (0.005)	
	Avg_post_negativity	Average post negativity, as calculated using the VADER^c^ library	0.1509		0.082		0.071 (0.042)		0.0519 (0.036)	
	*Male*	Ratio of words within the “male” category	0.1355		0.039		0.0011 (0.0013)		0.0007 (0.001)	
	Ratio_octile_8	Ratio of activities from 9 PM to midnight	0.1265		0.045		0.0231 (0.356)		0.1227 (0.188)	
	*Ingest*	Ratio of words within the “ingest” category	0.0627		−0.056		0.0003 (0.0007)		0.0014 (0.0018)	
	*Insight*	Ratio of words within the “insight” category	0.0516		0.053		0.0044 (0.004)		0.0035 (0.003)	
	*Power*	Ratio of words within the “power” category	0.0308		−0.058		0.0024 (0.0026)		0.0042 (0.0036)	
	*We*	Ratio of words within the “we” category	0.0196		−0.056		0.0001 (0.0002)		0.0002 (0.0004)	
	*Prep*	Ratio of words within the “prepositions” category	0.0117		0.063		0.0028 (0.0026)		0.0017 (0.0017)	
**Instagram**										
	Avg_post_readability	Average post readability, as measured using the SMOG index	0.761		−0.203		5.1018 (1.15)		6.2564 (1.638)	
	*Space*	Ratio of words within the “space” category	0.733		−0.147		0.0031 (0.0025)		0.0042 (0.0025)	
	*Affiliation*	Ratio of words within the “affiliation” category	0.6839		−0.181		0.0032 (0.0027)		0.0056 (0.0034)	
	*Friend*	Ratio of words within the “friend” category	0.5336		−0.159		0.0009 (0.0027)		0.0018 (0.0034)	
	*Female*	Ratio of words within the “female” category	0.4576		−0.168		0.0008 (0.001)		0.0019 (0.0023)	
	*Sad*	Ratio of words within the “sad” category	0.4554		0.113		0.0011 (0.0008)		0.0007 (0.0012)	
	*Quant*	Ratio of words within the “quantifier” category	0.4195		−0.118		0.0012 (0.0013)		0.0019 (0.0016)	
	Away	Ratio of “away” within the overall bag of words	0.4064		−0.105		0.0768 (0.276)		0.2505 (0.5)	
	*Assent*	Ratio of words within the “assent” category	0.3913		−0.102		0.0008 (0.0012)		0.0013 (0.0014)	
	Next	Ratio of “next” within the overall bag of words	0.3854		−0.12		0.0957 (0.267)		0.6466 (1.236)	

^a^SSD: schizophrenia spectrum disorder.

^b^SMOG: Simple Measure of Gobbledygook.

### Attributing Divergent Construct Validity of Models to Divergent Identities on the Web

What could explain the observed differences in construct validities of the intraplatform models? Early in this paper, we posited that these differences might stem from people’s identities being fragmented across different platforms. To situate that these divergent identities are indeed the drivers behind differential cross-platform model construct validities and, by extension, performance, we adopted a strategy to measure the differences within the extracted feature space between the investigated platforms for a given participant. As social media data for participants on all platforms are encoded via feature vectors in this study, we calculated the pairwise similarity between platform-specific data using cosine similarity [36]. More specifically, we calculated the average cosine similarity within participants between platforms and compared it with the average cosine similarity between participants within platforms for participants with SSD with data on all 3 platforms. Given that, even within the same social media platform, different people can have unique modes of expressing their identities, we used the latter as a baseline for assessing whether fragments of identities representing an individual across platforms diverge more or less than the divergence of identities between individuals.

We found that the average between-platform, within-participant cosine similarity was 0.3093 for Facebook-Twitter, 0.2304 for Facebook-Instagram, and 0.3905 for Twitter-Instagram. This was either lower than or similar to the average within-platform, between-participant cosine similarity for the investigated platforms: 0.5072 for Facebook, 0.5427 for Twitter, and 0.373 for Instagram. The same trend holds even when calculating the averages using data from both participants with SSD and healthy controls with data from all 3 platforms.

## Discussion

### Principal Findings

Our study aimed to measure the ability (or inability) of mental health classifiers to generalize across platforms and surface evidence of fragmented identities on social media among patients with SSD. Overall, we found that, across the board, models trained on data from social media platforms have poor generalizability when evaluated on data from other social media platforms even when holding the feature set constant across training and testing data. This trend holds true even in the 2 robustness tests, where the same participants and data set size were used in the training and testing data (as described in the Methods section). This trend is also true even when the training data come from a platform with high data availability and the testing data come from a platform with low data availability. For instance, the best *F*_1_-score of the intraplatform models for Twitter (0.63) was 0.257 (69%) higher compared with the best *F*_1_-score of the interplatform models for Twitter, where the training data came from Facebook (0.373).

Next, we discuss the findings regarding feature importance in more detail. First, looking at the theoretical validity of the top 10 features per platform and interpretation of the sign of the features’ logistic regression coefficient, we found alignment with previous literature and evidence of clinical meaningfulness [7,8,11]. For instance, given the positive coefficient from the trained logistic regression model presented in Table 7, higher levels of use of lexicon indicative of negative emotions are highly predictive of SSD for Facebook (see the example post in Textbox 3 highlighting words such as “fear,” “fail,” and “hurts”). This confirms literature noting that a reduced ability to feel or express pleasure (anhedonia) is common in patients with SSD [37]. Similarly, previous research has found anger-related terms commonly appearing in social media posts before the onset of early psychosis as well as preceding a psychiatric hospitalization [38]. This may explain why higher levels of use of lexicon indicative of the Linguistic Inquiry and Word Count category *Anger* are also highly predictive of SSD for Facebook (example post in Textbox 3 containing *Anger* words such as “shit” and “fucking”). Finally, words and phrases such as those in the Linguistic Inquiry and Word Count *Sad* category (eg, “useless,” “sorry,” and “sob”) point to typical negative symptoms of SSD [39]. They can be indicative of a decreased sense of purpose and a seeming lack of interest in the world [39]. Models trained on Instagram successfully picked up such cues from the posts, where higher use of such vocabulary was indicative of an impending psychiatric hospitalization because of SSD.

That said, each model corresponding to each platform seemed to pick up contrasting signals from its respective training data, which is why we note the low overlap in the aforementioned top SHAP features. Among the few that overlap in the top 10 features reported previously, we found “avg_post_readability” to be picked up as a highly predictive feature by both Facebook and Instagram models, whereas “ratio_octile8” was selected by both Facebook and Twitter models. In our case, “avg_post_readability” is calculated using the Simple Measure of Gobbledygook index, which approximates the years of education needed to fully comprehend a piece of written text. The negative logistic regression coefficient and the averages of the SSD and control groups for this feature suggest that texts written by patients with SSD are simpler in nature, which is indicative of language dysfunction. This is a known negative symptom of schizophrenia and related psychotic disorders, as observed in prior work [40]. In addition, higher levels of late-night activity such as web or social media use, captured in the “ratio_octile8” feature, have been known to be associated with deteriorated mental health [41]. Finally, we found significant divergence in the distribution of feature importance between the platforms, as indicated by the low pairwise Kendall τ (<0.1) for the platforms’ feature importance rankings. These qualitative and quantitative results broadly imply that the models were being trained on considerably different data sources with differing content and contexts of use, which likely contributed to poor cross-platform model generalization.

At the crux of these differences, we found that the models had inherently different construct validity across platforms. Data on each platform reflect only a segment of an individual’s identity—a segment that may be absent in another platform. The fragmentation of one’s identity on social media can be most clearly seen among participants with data on all 3 platforms. In the analysis presented at the end of the Results section, we found low average pairwise cosine similarities within participants between platforms, especially when comparing with cosine similarities of different participants within the same platform. This indicates that, even within the same feature space for the same participant, social media data between platforms are likely to diverge into multiple distinct directions mapping to these fragments of identities. This divergence is at least equal to, if not even greater than, the divergence in identity presentation between different individuals within the same social media platform. Therefore, when models trained on data from one platform learn this specific fragment of identity, they are less effective on testing data that capture a different identity.

**Example (paraphrased and deidentified) posts representative of example top features to distinguish between schizophrenia spectrum disorder and control classes. Words indicative of the features are italicized.**

**NegEmo**

- I *fear* to try and *fail*, because i don’t want to be part of the STATISTIC of people that *failed*. It *hurts* when the opportunity passes by though.’

**Swear**

- Omfg the Damn *mf* #struggle to stay the *fking* sleep I’m like *wtf* this isn’t fair I hate my Damn neck hurting like this *shit* isn’t cool this pain waking me up every Damn hr

**Sad**

- Im a *useless sorry sob*

**Anger**

- Yo stay *tf* out my room unless we *fucking* cause I’m tired too tired for this *shit* 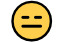 and all my *shit* better be where i left it

### Comparison With Prior Work

Our findings provide replicative validity to several threads in previous research. Specifically, we found that the performance of models trained on social media data with clinically verified labels (ie, SSD or control) is consistent with similar models presented in previous research, including those trained on similar patient populations and clinical sites [6,8]. Furthermore, linguistic differences reflecting serious mental health conditions between social media platforms found in our work have also been elucidated upon in previous work. For instance, Guntuku et al [42] found that there is little overlap between words indicative of stress on Twitter and Facebook. In addition, our findings regarding the low performance of models for interplatform tasks compared with intraplatform tasks follow a similar vein to those of the study by Ernala et al [8]. In their study, they found that, despite the overwhelming advantage in data availability, models trained on social media data with self-reported labels significantly underperformed models trained on social media data with clinically verified labels when evaluated on clinical testing data [8]. Similar to our experiments, such a difference in performance in the study by Ernala et al [8] was also noted to be caused by a mismatch in important features learned by the different models to differentiate between language and activity patterns deployed by patients with SSD and healthy controls. Overall, our analysis combined with previous results suggests that construct validities of predictive models trained on data from different social media platforms are dissimilar, reinforcing the need for continued exploration of novel social media–based early identification strategies with a special emphasis on uniting distinct fragments of identities for accurate identification and intervention.

### Clinical Implications

Our findings have important implications for mental health research and practice. Hospitalization prediction for psychiatric illnesses by harnessing digital trace data has been of significant interest in recent years. These previous studies have explored the utility of smartphone sensor data (ie, geolocation, physical activity, phone use, and speech), wearables, and social media activity to predict symptom fluctuations as well as understand the diagnostic process and hospitalization identification [6,43-46]. Our work extends this body of research by critically examining how machine learning efforts that harness data from single sources may not be readily applicable to support hospitalization prediction in contexts where the same source of data is not present. For these models to be usable in the real world, we advocate for a comprehensive approach in which clinicians look to patterns gleaned through the integration of different data sources while augmenting their decision-making with objective measures derived from digital trace data. Social media data are also increasingly becoming a part of consultations [47,48]. Therefore, we suggest that clinicians consider both acknowledging and incorporating collateral information spanning multiple platforms into the way they monitor symptomatic exacerbation in their patients and modify treatment to prevent further hospitalizations.

Finally, digital interventions that are touted to be powered by social media data should consider the significant aspect of fragmented web-based identities of patients [49,50]. To intervene at the right time, at the right place, and for the right person, a comprehensive approach to understanding a patient’s context for hospitalization prediction would be beneficial. However, we recognize that, in a domain as sensitive as mental health, combining data sources may further complicate the privacy and ethical risks to those who contribute their data—research has shown that information integration can enable the discovery of otherwise latent attributes, some of which may present grave feelings of discomfort and violation in individuals [51,52]. Therefore, we urge caution and call for new standards to protect the confidentiality and rights of this sensitive population and ensure that the enabled technologies are used in the service of positive outcomes for the patients.

### Limitations and Future Work

Our work has some limitations that could be addressed in future research. First, despite the use of data augmentation techniques to rebalance the ratio between SSD data and control data for each data set and make the data set sizes of the 3 examined platforms (ie, Instagram, Twitter, and Facebook) comparable with each other, we acknowledge that a limited quantity of available data may have affected the observed classification performance. Although it is widely recognized that patient social media data are challenging to collect, as was the case in this study, future research may consider the potential of creating large benchmarked data sets that may support better reproducible research in this field [53]. Second, we acknowledge the demographic dissimilarity between participants with SSD and healthy controls, which may be a confounding factor in our study design. Furthermore, our methods did not examine or extract any features concerning video data, which are available on Facebook and especially Instagram. Given that youths nowadays are increasingly expressing themselves on social media via videos (especially on video-centric platforms such as TikTok), future research should aim to fill these gaps so that we can ensure the completeness of one’s mental health records expressed on social media and other forms of networked communication. Along these lines, future research may also consider data from additional novel social media platforms that are increasingly being used by youths for their social goals, such as Snapchat and TikTok. Finally, it would be worthwhile to examine additional clinical questions such as suicidal risk to explore the extent to which identity fragmentation across social media platforms may affect the quality of inferences made from these data.

### Conclusions

In this study, we showed that it is challenging to build effective models for predicting future psychiatric hospitalizations of patients with SSD on new social media data from platforms previously unseen in the models’ training data. Specifically, we demonstrated that models built on one platform’s data do not generalize to another as each platform consistently reflects different segments of participants’ identities. This fragmentation of identity is empirically backed up by both significant differences in the construct validity of intraplatform classifiers and divergent feature vectors within participants between the 3 investigated social media platforms. To ensure the effective incorporation of digital technology into early psychosis intervention, especially in the prevention of relapse hospitalizations, further research must explore precisely how symptoms of mental illness manifest on the web through changing patterns of language and activity on various platforms as well as how comprehensive, ethical, and effective treatment and engagement strategies should be devised that function seamlessly across patients’ fragmented web-based identities.

## Appendix

Multimedia Appendix 1 Additional information on the feature selection process and robustness checks.

## Abbreviations

AUROC: area under the receiver operating characteristic curve
SHAP: Shapley Additive Explanations
SSD: schizophrenia spectrum disorder

## References

[1] Wolthaus JE, Dingemans PM, Schene AH, Linszen DH, Wiersma D, Van Den Bosch RJ, Cahn W, Hijman R (2002). Caregiver burden in recent-onset schizophrenia and spectrum disorders: the influence of symptoms and personality traits. J Nerv Ment Dis.

[2] Birchwood M, Macmillan F (1993). Early intervention in schizophrenia. Aust N Z J Psychiatry.

[3] Lieberman JA, Fenton WS (2000). Delayed detection of psychosis: causes, consequences, and effect on public health. Am J Psychiatry.

[4] Birnbaum ML, Rizvi AF, Confino J, Correll CU, Kane JM (2017). Role of social media and the internet in pathways to care for adolescents and young adults with psychotic disorders and non-psychotic mood disorders. Early Interv Psychiatry.

[5] Miller BJ, Stewart A, Schrimsher J, Peeples D, Buckley PF (2015). How connected are people with schizophrenia? Cell phone, computer, email, and social media use. Psychiatry Res.

[6] Birnbaum ML, Ernala SK, Rizvi AF, Arenare E, R Van Meter A, De Choudhury M, Kane JM (2019). Detecting relapse in youth with psychotic disorders utilizing patient-generated and patient-contributed digital data from Facebook. NPJ Schizophr.

[7] Mitchell M, Hollingshead K, Coppersmith G (2015). Quantifying the language of schizophrenia in social media. Proceedings of the 2nd Workshop on Computational Linguistics and Clinical Psychology: From Linguistic Signal to Clinical Reality.

[8] Ernala S, Birnbaum M, Candan K, Rizvi A, Sterling W, Kane J, De Choudhury M (2019). Methodological gaps in predicting mental health states from social media: triangulating diagnostic signals. Proceedings of the 2019 CHI Conference on Human Factors in Computing Systems.

[9] Rekhi G, Ang MS, Lee J (2019). Clinical determinants of social media use in individuals with schizophrenia. PLoS One.

[10] Zomick J, Levitan S, Serper M (2019). Linguistic analysis of schizophrenia in Reddit posts. Proceedings of the Sixth Workshop on Computational Linguistics and Clinical Psychology.

[11] Birnbaum ML, Ernala SK, Rizvi AF, De Choudhury M, Kane JM (2017). A collaborative approach to identifying social media markers of schizophrenia by employing machine learning and clinical appraisals. J Med Internet Res.

[12] Ernala SK, Rizvi AF, Birnbaum ML, Kane JM, De Choudhury M (2017). Linguistic markers indicating therapeutic outcomes of social media disclosures of schizophrenia. Proc ACM Human Comput Interact.

[13] Auxier B, Anderson M (2021). Social media use in 2021. Pew Research Center.

[14] Hänsel K, Lin IW, Sobolev M, Muscat W, Yum-Chan S, De Choudhury M, Kane JM, Birnbaum ML (2021). Utilizing Instagram data to identify usage patterns associated with schizophrenia spectrum disorders. Front Psychiatry.

[15] Birnbaum ML, Norel R, Van Meter A, Ali AF, Arenare E, Eyigoz E, Agurto C, Germano N, Kane JM, Cecchi GA (2020). Identifying signals associated with psychiatric illness utilizing language and images posted to Facebook. NPJ Schizophr.

[16] Chancellor S, De Choudhury M (2020). Methods in predictive techniques for mental health status on social media: a critical review. NPJ Digit Med.

[17] Kircaburun K, Griffiths MD (2018). Instagram addiction and the big five of personality: the mediating role of self-liking. J Behav Addict.

[18] Bayer JB, Ellison NB, Schoenebeck SY, Falk EB (2015). Sharing the small moments: ephemeral social interaction on Snapchat. Inform Commun Soc.

[19] Purwaningtyas MP, Alicya DA (2020). The fragmented self: having multiple accounts in Instagram usage practice among Indonesian youth. J Media dan Komunikasi Indonesia.

[20] Gündüz U (2017). The effect of social media on identity construction. Mediterranean J Social Sci.

[21] First M, Spitzer RL, Gibbon M, Williams J (2002). Structured Clinical Interview for DSM-IV-TR Axis I Disorders, Research Version.

[22] Zimmerman M, Mattia JI (2001). A self-report scale to help make psychiatric diagnoses: the psychiatric diagnostic screening questionnaire. Arch Gen Psychiatry.

[23] Ernala SK, Kashiparekh KH, Bolous A, Ali A, Kane J, Birnbaum ML, DE Choudhury M (2021). A social media study on mental health status transitions surrounding psychiatric hospitalizations. Proc ACM Hum Comput Interact.

[24] Aizawa A (2003). An information-theoretic perspective of tf–idf measures. Inform Process Manag.

[25] Tausczik YR, Pennebaker JW (2009). The psychological meaning of words: LIWC and computerized text analysis methods. J Language Social Psychol.

[26] Mclaughlin G (1969). SMOG grading - a new readability formula. J Reading.

[27] Hutto C, Gilbert E (2014). VADER: a parsimonious rule-based model for sentiment analysis of social media text. Proceedings of the International AAAI Conference on Web and Social Media.

[28] Garimella V, Alfayad A, Weber I (2016). Social media image analysis for public health. Proceedings of the 2016 CHI Conference on Human Factors in Computing Systems.

[29] Chawla NV, Bowyer KW, Hall LO, Kegelmeyer WP (2002). SMOTE: synthetic minority over-sampling technique. J Artif Intell Res.

[30] Guyon I, Elisseeff A (2003). An introduction to variable and feature selection. J Mach Learn Res.

[31] Pedregosa F, Varoquaux G, Gramfort A, Michel V, Thirion B, Grisel O, Blondel M, Prettenhofer P, Weiss R, Dubourg V, Vanderplas J, Passos A, Cournapeau D, Brucher M, Perrot M, Duchesnay E (2011). Scikit-learn: machine learning in Python. JMLR.

[32] Hastie T, Tibshirani R, Friedman J (2009). The Elements of Statistical Learning Data Mining, Inference, and Prediction.

[33] Powers DM (2020). Evaluation: from precision, recall and F-measure to ROC, informedness, markedness and correlation. Int J Mach Learn Technol.

[34] Lundberg S, Lee S (2017). A unified approach to interpreting model predictions. arXiv.

[35] (1992). Handbook of Game Theory with Economic Applications Volume 2.

[36] Jurafsky D, Martin JH (2000). Speech and Language Processing An Introduction to Natural Language Processing, Computational Linguistics, and Speech Recognition.

[37] Kwapil TR (1998). Social anhedonia as a predictor of the development of schizophrenia-spectrum disorders. J Abnormal Psychol.

[38] Ringer JM, Lysaker PH (2014). Anger expression styles in schizophrenia spectrum disorders: associations with anxiety, paranoia, emotion recognition, and trauma history. J Nerv Ment Dis.

[39] Liu J, Chua JJ, Chong SA, Subramaniam M, Mahendran R (2020). The impact of emotion dysregulation on positive and negative symptoms in schizophrenia spectrum disorders: a systematic review. J Clin Psychol.

[40] Kuperberg G, Caplan D (2003). Language dysfunction in schizophrenia. Neuropsychiatry.

[41] Palmese LB, DeGeorge PC, Ratliff JC, Srihari VH, Wexler BE, Krystal AD, Tek C (2011). Insomnia is frequent in schizophrenia and associated with night eating and obesity. Schizophr Res.

[42] Chandra Guntuku S, Buffone A, Jaidka K, Eichstaedt JC, Ungar LH (2019). Understanding and measuring psychological stress using social media. Proceedings of the International AAAI Conference on Web and Social Media.

[43] Ben-Zeev D, Scherer EA, Wang R, Xie H, Campbell AT (2015). Next-generation psychiatric assessment: using smartphone sensors to monitor behavior and mental health. Psychiatr Rehabil J.

[44] Birnbaum ML, Kulkarni P", Van Meter A, Chen V, Rizvi AF, Arenare E, De Choudhury M, Kane JM (2020). Utilizing machine learning on internet search activity to support the diagnostic process and relapse detection in young individuals with early psychosis: feasibility study. JMIR Ment Health.

[45] Eisner E, Bucci S, Berry N, Emsley R, Barrowclough C, Drake RJ (2019). Feasibility of using a smartphone app to assess early signs, basic symptoms and psychotic symptoms over six months: a preliminary report. Schizophr Res.

[46] Zulueta J, Piscitello A, Rasic M, Easter R, Babu P, Langenecker SA, McInnis M, Ajilore O, Nelson PC, Ryan K, Leow A (2018). Predicting mood disturbance severity with mobile phone keystroke metadata: a BiAffect digital phenotyping study. J Med Internet Res.

[47] Fisher CE, Appelbaum PS (2017). Beyond googling: the ethics of using patients' electronic footprints in psychiatric practice. Harv Rev Psychiatry.

[48] Rieger A, Gaines A, Barnett I, Baldassano CF, Connolly Gibbons MB, Crits-Christoph P (2019). Psychiatry outpatients’ willingness to share social media posts and smartphone data for research and clinical purposes: survey study. JMIR Form Res.

[49] Yoo D, Birnbaum M, Van Meter A, Ali A, Arenare E, Abowd G, De Choudhury M (2020). Designing a clinician-facing tool for using insights from patients’ social media activity: iterative co-design approach. JMIR Ment Health.

[50] Yoo D, Ernala S, Saket B, Weir D, Arenare E, Ali A, Van Meter Ar, Birnbaum Ml, Abowd Gd, De Choudhury M (2021). Clinician perspectives on using computational mental health insights from patients’ social media activities: design and qualitative evaluation of a prototype. JMIR Ment Health.

[51] Terrasse M, Gorin M, Sisti D (2019). Social media, e‐health, and medical ethics. Hastings Center Report.

[52] Thieme A, Belgrave D, Sano A, Doherty G (2020). Machine learning applications. Interactions.

[53] Househ M, Grainger R, Petersen C, Bamidis P, Merolli M (2018). Balancing between privacy and patient needs for health information in the age of participatory health and social media: a scoping review. Yearb Med Inform.

