# Supplementary material for: Cross-Platform Detection of Psychiatric Hospitalization via Social Media Data: Comparison Study
Source: JMIR Ment Health. 2022 Dec 30;9(12):e39747. doi: 10.2196/39747 (PMC9840099; doi:10.2196/39747)
Supplement: Multimedia Appendix 1 [file mental_v9i12e39747_app1.docx]

**Multimedia Appendix 1**

**CROSS-PLATFORM DETECTION OF PSYCHIATRIC HOSPITALIZATION VIA SOCIAL MEDIA DATA: A COMPARISON STUDY**

**Supplementary Information**

Cuong Viet Nguyen, Nathaniel Lu, John M. Kane, Michael L. Birnbaum, Munmun De Choudhury

- **More details on feature selection**

To encode participant’s social media data for downstream classification and analysis tasks outlined in our research objectives, we identified and extracted the following categories of features from these data for all three of the investigated social platforms. The specific feature categories were chosen based on relevant prior literature, particularly relating to the use of social media data to infer mental health attributes and psychiatric outcomes [7, 12]. Note that all features were computed at the individual participant level:

- *n-gram language features* (d = 500): A language model of 500 top unigrams and

bigrams was generated from the entire textual windowed data of participants from a given social media platform. The features in this category represent the term frequency-inverse document frequency (tf-idf) frequency [24] counts of those top n-grams.

- *Linguistic Inquiry and Word Count (LIWC)* (d = 78): The widely validated

LIWC lexicon [25] was employed, which identifies linguistic measures for the following psycholinguistic categories in the textual data of the participants: (1) affective attributes, including positive and negative affect, anger, anxiety, sadness, swearing; (2) cognitive attributes, including both cognition categories comprising of cognitive mechanisms, discrepancies, inhibition, negation, causation, certainty, and tentativeness, and perception categories comprising of see, hear, feel, percept, insight, and relative; and (3) linguistic style attributes, including lexical density (verbs, auxiliary verbs, adverbs, prepositions, conjunctions, articles, inclusive, and exclusive), temporal references (past, present, and future tenses), social/personal concerns (family, friends, social, work, health). Each feature here represents the normalized frequency of words belonging to each LIWC category.

- *Lexico-semantic features* (d = 3): These features encode summaries on lexical and semantic information given the entirety of participants’ textual data on a given social media platform that has not been encoded by the previous two categories of features. These include the average readability (calculated using the Simple Measure of Gobbledygook (SMOG) index [26]), the average length of posts, and the average Valence Aware Dictionary for sEntiment Reasoning (VADER) sentiment polarity score [27].
- *Activity features* (d = 9): These features encode the participants’ posting pattern on the respective social media platforms. They include the average number of activities per day (only counting days when the participant is active on that platform), and the ratio between the count of activities done during each octile of the day and the total activity count.
- *Image features* (d = 23, Instagram and Facebook only): These features encode the various information available from the participants’ image data in the respective social media platforms, including but not limited to the average colorfulness and the average number of faces [28]. We particularly referenced features that have previously been deployed to distinguish images belonging to participants with SSD and participants within the healthy control group [14].
- **Robustness Test 1 – Platform Data Availability**

To ensure that changes in classifiers’ performances between the intra-platform and inter-platform experiments were not caused by the presence of participants with only data from one platform within datasets deployed in the inter-platform experiments, we performed a robustness check where we re-ran the inter-platform experiments as described above, but now both the training and testing data only including participants with data for both the training and testing platform.

For inter-platform with only dual-platform users, the average F1 score slightly increases compared to results from the original experiments at 0.474 F1. Still, even with these gains, the best models achieve prediction performance nowhere close to the intraplatform experiments. Results from this test is presented in Table S1

- **Robustness Test 2 - Dataset Size**

In addition, to also ensure that differences in classifiers’ performances between platforms for both intra-platform and inter-platform experiments were not primarily driven by the disparity in dataset size between the platforms and classes, we performed another robustness check to see if such differences remain after the dataset sizes are equalized via data augmentation techniques. We first rebalanced all three datasets so that the ratio between control participants and participants with SSD is 1:1 utilizing the Synthetic Minority Oversampling Technique (SMOTE) [29]. This method tackles class imbalance in classification tasks. In this way, we increased the training dataset sizes of the Twitter and Instagram datasets to be similar to that of the Facebook dataset by creating synthetic participants from existing participants using the same SMOTE process because Facebook had the most data. Finally, we run the same intra- and inter-platform experiments with the rebalanced datasets and record the results.

For both intra- and inter-platform experiments with equal training dataset sizes, the average F1 score decreases slightly to 0.646 for intra-platform experiments and increases slightly to 0.473 for inter-platform experiments. Like Robustness Test 1, while there is a small increase in performance resulting from holding users consistent across platforms, the best models still achieve prediction performance nowhere close to the intraplatform experiments. Results from this test are presented in Table S2

- **Robustness Test 3 – Combining Insights from Multiple Platforms**

Finally, we test whether combining datasets of three platforms into a single dataset can ameliorate the differences in classifiers’ performances between platforms. Here, we split each of the datasets into training and test sets with an 80:20 ratio using the same technique as the intra-platform experiments. Then, we combine the training sets of all three platforms into a single training dataset, with platform as an additional feature, and use it to train “unified” classification models containing insights from all three platforms. We assume that data from different platforms of the same participant are independent observations for model training purposes. Finally, we test these models on the test sets for all three platforms and record the results.

By performing this test, we did find a small improvement of 0.5% in the average performance of the Logistic Regression on the Facebook, Twitter, and Instagram test-set, going from 0.72 F1 for the original training data to 0.724 F1 for the combined training dataset. Results from this test are presented in Table S3

**Table S1:** Results from the Robustness Test 1

|  | **Facebook – Twitter** | | | | | **Facebook – Instagram** | | | | |
| --- | --- | --- | --- | --- | --- | --- | --- | --- | --- | --- |
| Models | Acc | P | R | F1 | AUROC | Acc | P | R | F1 | AUROC |
| Random Forest | 0.891 | 0.0 | 0 | 0.0 | 0.5 | 0.634 | 0.353 | 0.387 | 0.369 | 0.557 |
| SVM | 0.848 | 0.0 | 0.0 | 0.0 | 0.476 | 0.411 | 0.278 | 0.710 | 0.4 | 0.503 |
| MLP | 0.783 | 0.143 | 0.2 | 0.2 | 0.527 | 0.482 | 0.292 | 0.613 | 0.396 | 0.522 |
| Logistic Regression | 0.848 | 0.25 | 0.2 | 0.2 | 0.563 | 0.616 | 0.385 | 0.645 | 0.482 | 0.625 |

**Table S2:** Results from Robustness Test 2

|  | **Facebook** | | | | **Twitter** | | | | **Instagram** | | | |
| --- | --- | --- | --- | --- | --- | --- | --- | --- | --- | --- | --- | --- |
| Models | Acc | P | R | F1 | Acc | P | R | F1 | Acc | P | R | F1 |
| Random Forest | 0.718 | 0.731 | 0.72 | 0.715 | 0.9 | 0.45 | 0.5 | 0.474 | 0.738 | 0.716 | 0.651 | 0.653 |
| SVM | 0.726 | 0.728 | 0.726 | 0.725 | 0.86 | 0.447 | 0.478 | 0.461 | 0.838 | 0.82 | 0.814 | 0.809 |
| MLP | 0.522 | 0.507 | 0.528 | 0.395 | 0.9 | 0.45 | 0.5 | 0.474 | 0.7 | 0.683 | 0.616 | 0.574 |
| Logistic Regression | 0.743 | 0.749 | 0.744 | 0.741 | 0.88 | 0.449 | 0.489 | 0.468 | 0.785 | 0.743 | 0.733 | 0.730 |

**Table S3:** Results from Robustness Test 3

|  | **Facebook** | | | | **Twitter** | | | | **Instagram** | | | |
| --- | --- | --- | --- | --- | --- | --- | --- | --- | --- | --- | --- | --- |
| Models | Acc | P | R | F1 | Acc | P | R | F1 | Acc | P | R | F1 |
| Random Forest | 0.669 | 0.673 | 0.669 | 0.668 | 0.76 | 0.586 | 0.6 | 0.565 | 0.754 | 0.758 | 0.649 | 0.653 |
| SVM | 0.694 | 0.702 | 0.692 | 0.690 | 0.74 | 0.593 | 0.677 | 0.584 | 0.715 | 0.674 | 0.676 | 0.672 |
| MLP | 0.735 | 0.741 | 0.733 | 0.731 | 0.8 | 0.608 | 0.711 | 0.623 | 0.761 | 0.730 | 0.724 | 0.719 |
| Logistic Regression | 0.763 | 0.764 | 0.760 | 0.758 | 0.84 | 0.611 | 0.733 | 0.637 | 0.789 | 0.765 | 0.803 | 0.776 |
